# Clinical diagnosis and treatment analysis of 11 cases of unicentric Castleman disease in the retroperitoneum

**DOI:** 10.3389/fonc.2026.1750000

**Published:** 2026-01-26

**Authors:** Ke Dong, Hongyu Zhang, Lunan Wu, Jie Liu

**Affiliations:** 1 Department of General Surgery, Jiaozhou Central Hospital, Qingdao, China; 2 Department of Thoracic Surgery, Qingdao Women and Children's Hospital, Qingdao, China; 3 Department of General Surgery, Peking University People’s Hospital Qingdao Hospital, Qingdao, China

**Keywords:** Castleman disease, prognosis, rare, retroperitoneal neoplasms, surgery

## Abstract

**Objective:**

To investigate the diagnosis, treatment, and prognosis of unicentric Castleman disease (UCD) located in the retroperitoneum.

**Methods:**

A retrospective analysis was conducted on the clinical data of 11 patients with pathologically confirmed retroperitoneal UCD at Peking University People’s Hospital between 2010 and 2025. Data collected included gender, age, clinical manifestations, routine blood tests, tumor markers, treatment modalities, pathological characteristics, and treatment outcomes. Postoperative survival status was assessed via outpatient records and telephone follow-up.

**Results:**

The cohort comprised 3 males and 8 females. One male patient had a history of long-term smoking and alcohol use, while the other 10 patients had no such history. One patient presented with lower back discomfort, one with abdominal discomfort, and the remaining 9 patients were incidentally diagnosed during health check-ups. All patients had serum inflammatory markers and tumor markers within normal ranges. Preoperative abdominal contrast-enhanced CT/MRI localized the lesions in all cases. The mean maximum tumor diameter was 5.23 ± 2.05 cm. Five patients underwent laparoscopic surgery, and six underwent open surgery. The mean operative time was 2.73 ± 1.21 hours, with an intraoperative blood loss of 100 ml (IQR 20–100 ml). No severe postoperative complications occurred. The mean hospital stay was 5.00 ± 1.79 days, and all patients recovered well and were discharged. Pathological examination revealed the hyaline vascular type in 9 cases, the plasma cell type in 1 case, and the mixed type in 1 case. The mean follow-up duration was 68.00 ± 30.41 months (range: 25–112 months). By the end of follow-up, no recurrence was observed, and all patients remained alive and healthy.

**Conclusion:**

Retroperitoneal unicentric Castleman disease is rare and lacks specific clinical manifestations. Complete surgical resection is the optimal treatment, and patients exhibit excellent long-term prognosis upon follow-up.

## 1 Introduction

Castleman disease (CD) is a rare lymphoproliferative disorder with characteristic histopathological features, also known as giant lymph node hyperplasia or angiofollicular lymph node hyperplasia (1). It can occur in lymph node regions throughout the body, with the retroperitoneum being a notable site of involvement, accounting for approximately 17% of cases in a large surgical series (2). Clinically, CD is classified based on the number of affected lymph node regions into unicentric CD (UCD), involving a single region, and multicentric CD (MCD). Surgical resection is the cornerstone treatment for UCD (3). Previous literature consists mainly of case reports. This study retrospectively analyzed the clinical data of 11 patients with pathologically confirmed retroperitoneal UCD at Peking University People’s Hospital from 2010 to 2025, aiming to enhance understanding of this disease and provide reference for clinical diagnosis and management.

## 2 Materials and methods

### 2.1 Patient population

We retrospectively analyzed the clinical data of 11 patients with pathologically confirmed retroperitoneal UCD at Peking University People’s Hospital between 2010 and 2025. Inclusion criteria were: (1) pathological diagnosis consistent with CD; (2) disease confined to a single lymph node region in the retroperitoneum. Exclusion criteria were: (1) concurrent underlying diseases potentially causing Castleman-like lymph node changes; (2) incomplete clinical data. The study cohort included 3 males and 8 females, with a mean age of 49.09 ± 5.21 years. This study was approved by the Ethics Committee of Peking University People’s Hospital (Approval No:2025PHQDB028-01), and informed consent was obtained from all patients or their families.

### 2.2 Observation indicators and follow-up

Recorded parameters included gender, age, clinical manifestations, routine blood tests, tumor markers, treatment modalities, pathological features, and outcomes. Postoperative survival status was followed up via outpatient records and telephone interviews until April 2025.

### 2.3 Statistical analysis

Statistical analysis was performed using SPSS software (version 24.0). Normally distributed continuous data are presented as mean ± standard deviation (X ± s); non-normally distributed continuous data are presented as median (interquartile range, Q1, Q3).

## 3 Results

### 3.1 Baseline characteristics

Among the 11 patients, there were 3 males and 8 females. One male patient had a history of long-term smoking and alcohol use, while the other 10 patients had no such history. One patient presented with lower back discomfort, one with abdominal discomfort, and the remaining 9 patients were incidentally diagnosed during routine physical examinations. All patients had serum inflammatory markers and tumor markers within normal limits. Detailed baseline characteristics are shown in **Tables 1**, **2**.

**Table 1:** Baseline characteristics of 11 patients with retroperitoneal UCD.

Case no.	Gender	Age (years)	BMI(Kg /m^2^)	Comorbidities	Smoking history	Alcohol history	Chief complaint	Clinical type
1	Male	43	29.7	NO	NO	NO	Incidental finding	Unicentric
2	Female	50	23.8	NO	NO	NO	Incidental finding	Unicentric
3	Female	48	32.3	Ventricular premature beats	NO	NO	Incidental finding	Unicentric
4	Female	50	27.3	Hypertension	NO	NO	Incidental finding	Unicentric
5	Male	38	29.0	NO	NO	NO	Low back discomfort	Unicentric
6	Male	55	29.4	NO	Yes	Yes	Incidental finding	Unicentric
7	Female	50	20.3	NO	NO	NO	Incidental finding	Unicentric
8	Female	46	25.3	NO	NO	NO	Incidental finding	Unicentric
9	Female	55	25.7	NO	NO	NO	Abdominal discomfort	Unicentric
10	Female	54	26.1	NO	NO	NO	Incidental finding	Unicentric
11	Female	51	22.2	HBV carrier	NO	NO	Incidental finding	Unicentric

**Table 2:** Laboratory findings of 11 cases of retroperitoneal UCD.

Case no.	White blood cells (×10^9^/L)	C-reactive protein (mg/L)	Hemoglobin (g/L)	Erythrocyte sedimentation rate (mm/h)	Carcinoembryonic antigen (ng/mL)	Carbohydrate antigen 19-9 (U/mL)	HIV antibody
1	5.8	2.1	138	8	1.2	15	Negative
2	6.3	1.5	145	6	2.0	22	Negative
3	7.1	3.0	125	12	0.9	18	Negative
4	5.2	0.8	152	4	1.8	12	Negative
5	6.9	4.2	142	9	2.5	26	Negative
6	4.9	2.7	135	10	1.5	19	Negative
7	7.5	1.9	148	5	3.1	24	Negative
8	5.6	5.0	130	14	2.2	30	Negative
9	6.0	3.3	140	7	1.0	16	Negative
10	7.8	0.5	155	3	4.0	28	Negative
11	5.4	2.8	128	11	1.6	21	Negative

### 3.2 Perioperative data

**Figure 1:**
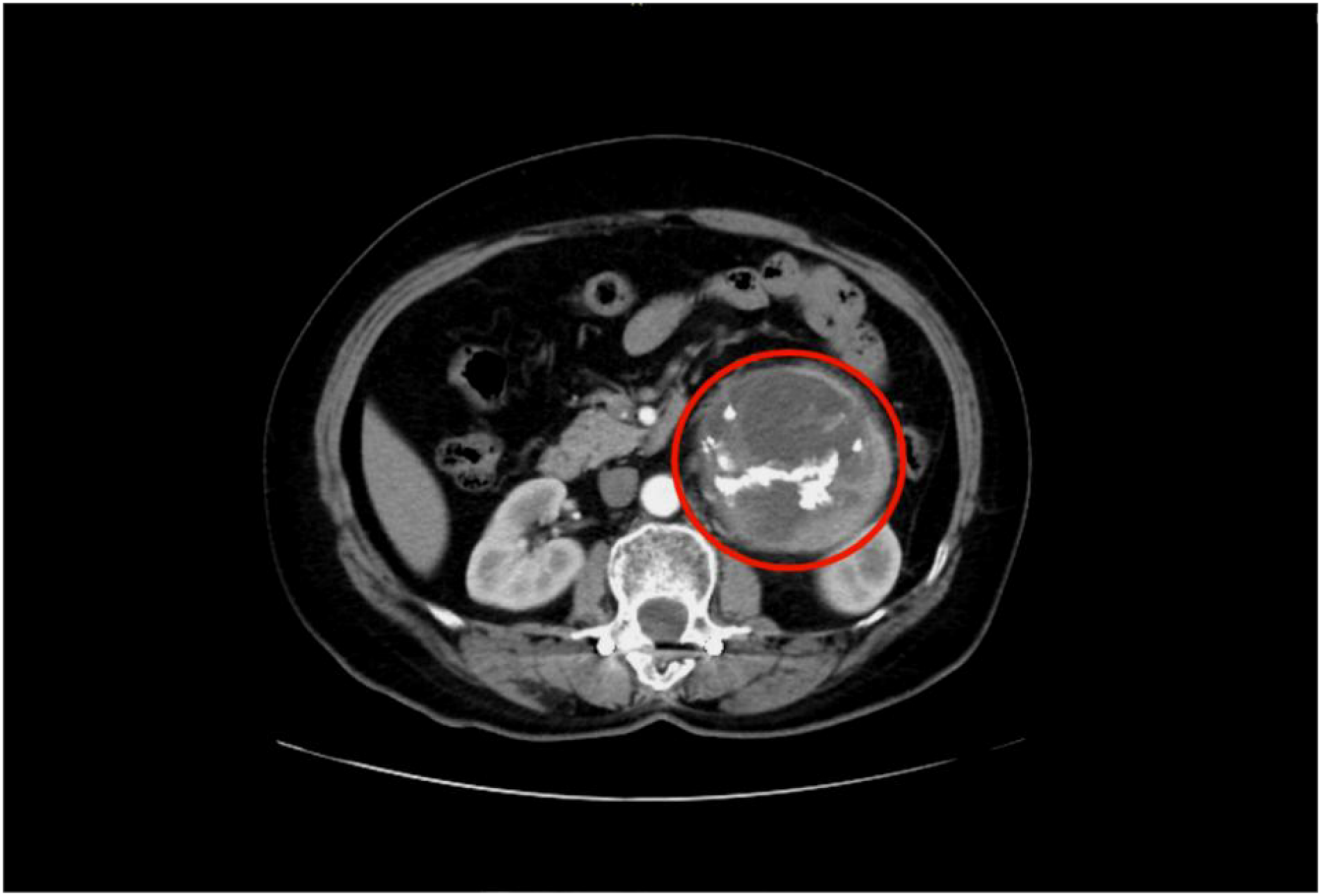
Retroperitoneal nodular mass with a well-defined border and intact capsule, showing internal calcification.

All 11 patients underwent preoperative localization of the lesion via abdominal contrast-enhanced CT/MRI; specific locations are listed in **Table 3**. The mean maximum tumor diameter was 5.23 ± 2.05 cm, with one patient having a maximum diameter of 10 cm. A representative imaging figure is shown in **Figure 1**. Five patients underwent laparoscopic surgery, and six underwent open surgery. The mean operative time was 2.73 ± 1.21 hours, with a median intraoperative blood loss of 100 ml (IQR 20–100 ml). No severe postoperative complications occurred. The mean postoperative hospital stay was 5.00 ± 1.79 days. All patients recovered successfully and were discharged.

**Table 3:** Perioperative data of 11 patients with retroperitoneal UCD.

Case no.	Imaging modality	Tumor location	Max tumor diameter (cm)	Preoperative diagnosis	Surgical approach	Operative time (hours)	Estimated blood loss (ml)	Postop complications	Postop hospital stay (days)
1	Dynamic Contrast-enhanced Abdominal CT	Left adrenal gland area	5.3	Adrenal tumor	Open	5h	100	None	7
2	Dynamic Contrast-enhanced Abdominal CT	Below left renal hilum	4.2	Retroperitoneal mass	Open	3h	100	None	5
3	Dynamic Contrast-enhanced Abdominal CT	Lower midline, left of abdominal aorta, posterior to inferior mesenteric artery	3.5	Paraganglioma	Laparoscopic	3h	20	None	3
4	Contrast-enhanced Lower Abdominal CT	Left adrenal gland area	4	Adrenal tumor	Laparoscopic	1h	10	None	4
5	Contrast-enhanced Abdominal MR	Between abdominal aorta and inferior vena cava	4.5	Retroperitoneal mass	Open	2h	20	None	6
6	Dynamic Contrast-enhanced Abdominal CT	Right adrenal gland area	5.5	Benign adrenal tumor	Laparoscopic	3h	100	None	4
7	Dynamic Contrast-enhanced Upper/Lower Abdominal CT	Anterior to inferior vena cava	4.5	Retroperitoneal mass	Laparoscopic	1h	20	None	3
8	Contrast-enhanced Upper Abdominal CT	Left upper quadrant, extending into left thoracic cavity, posterior to diaphragm, inferior to pancreatic upper border, medial to left of abdominal aorta	10	Retroperitoneal mass	Open	4h	800	None	9
9	Contrast-enhanced Lower Abdominal CT	Anterior to left kidney, adherent to left renal vessels and left ureter	8	Retroperitoneal mass	Laparoscopic	3.5h	200	None	5
10	Contrast-enhanced Pelvic CT	Lateral to left iliac vessels	5	Schwannoma	Open	2.5h	100	None	5
11	Contrast-enhanced Upper Abdominal CT	Right upper quadrant, between inferior liver pole and descending duodenum, adjacent to hepatic flexure of colon	3	Retroperitoneal mass	Open	2h	10	None	4

### 3.3 Pathological results and prognosis

**Figure 2:**
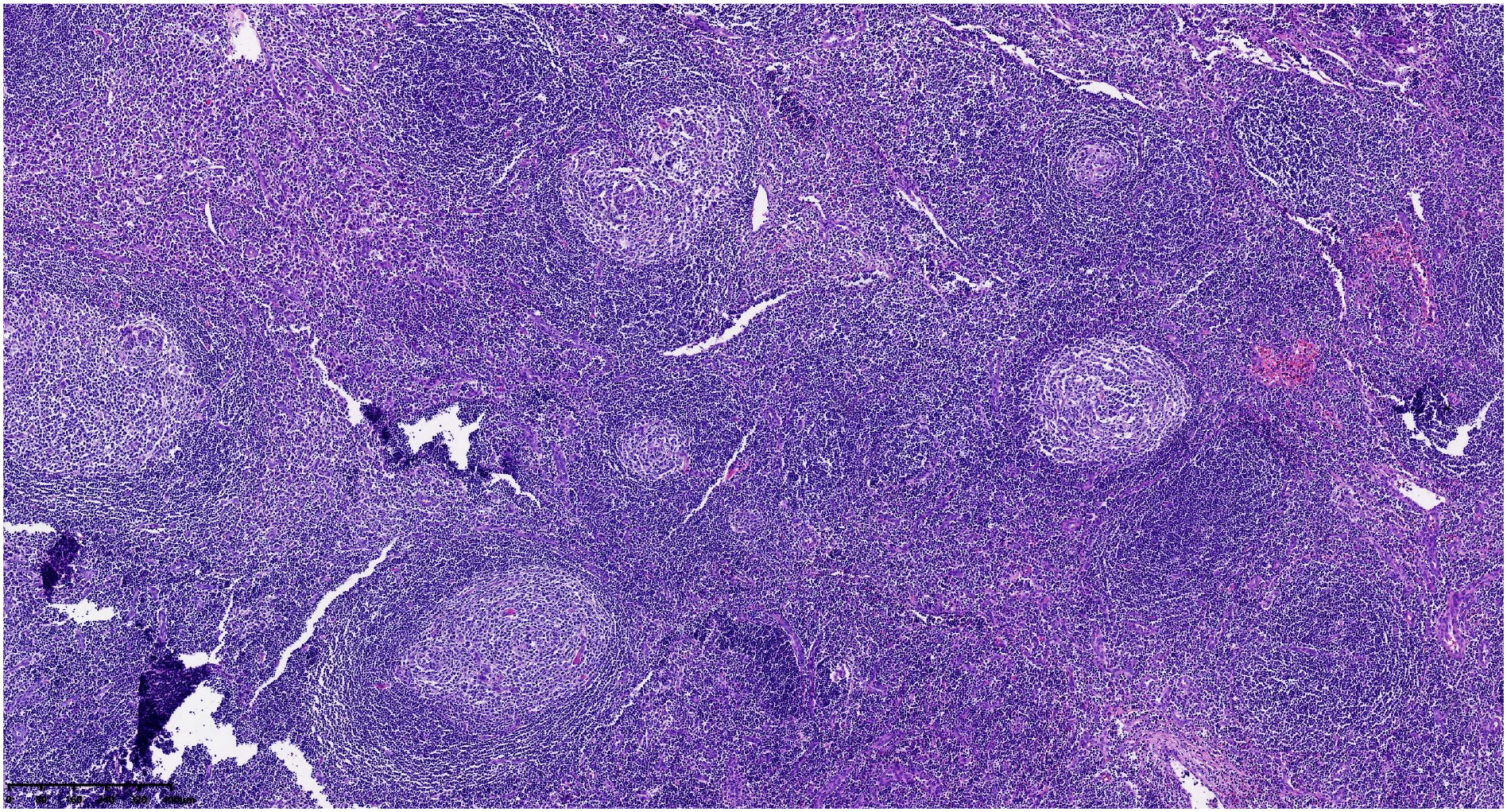
Atrophic germinal centers with concentrically arranged mantle zone cell proliferation (onion−skin appearance).

**Figure 3:**
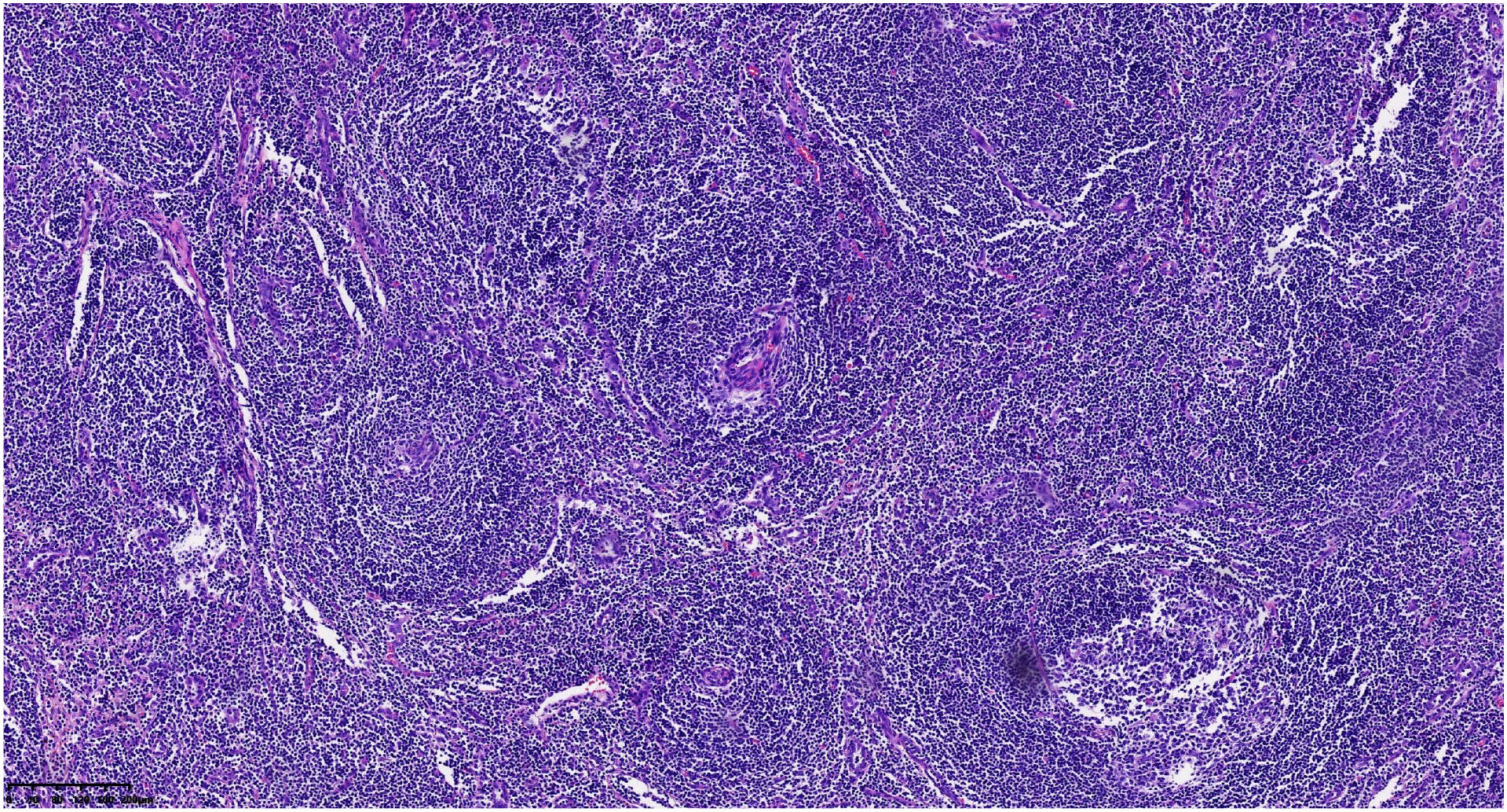
Follicles show atrophic germinal centers, ingrowth of small vessels, and expansile hyperplasia of monotonous−appearing mantle zone cells.

**Figure 4:**
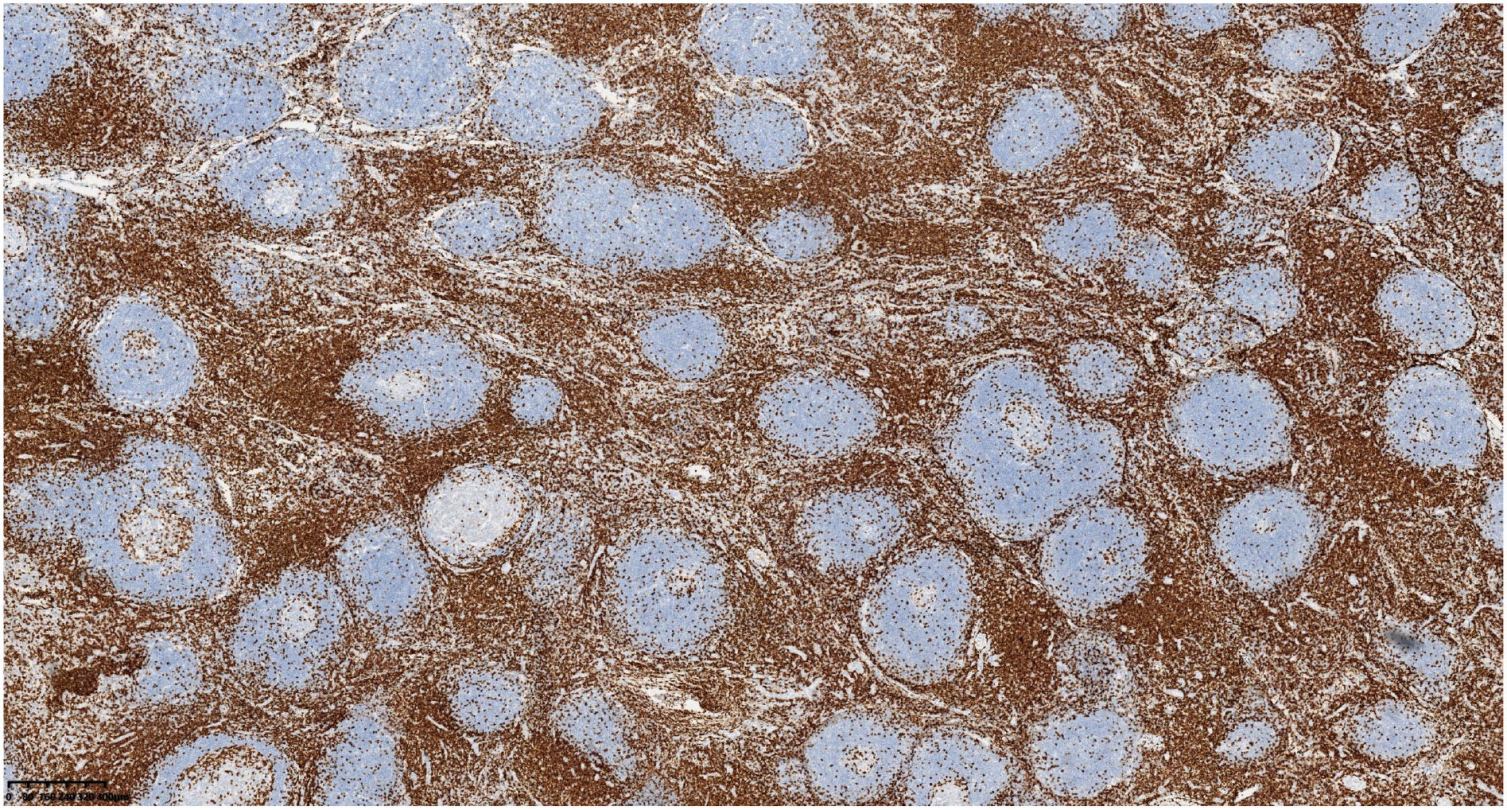
Interfollicular areas are positive for CD3.

**Figure 5:**
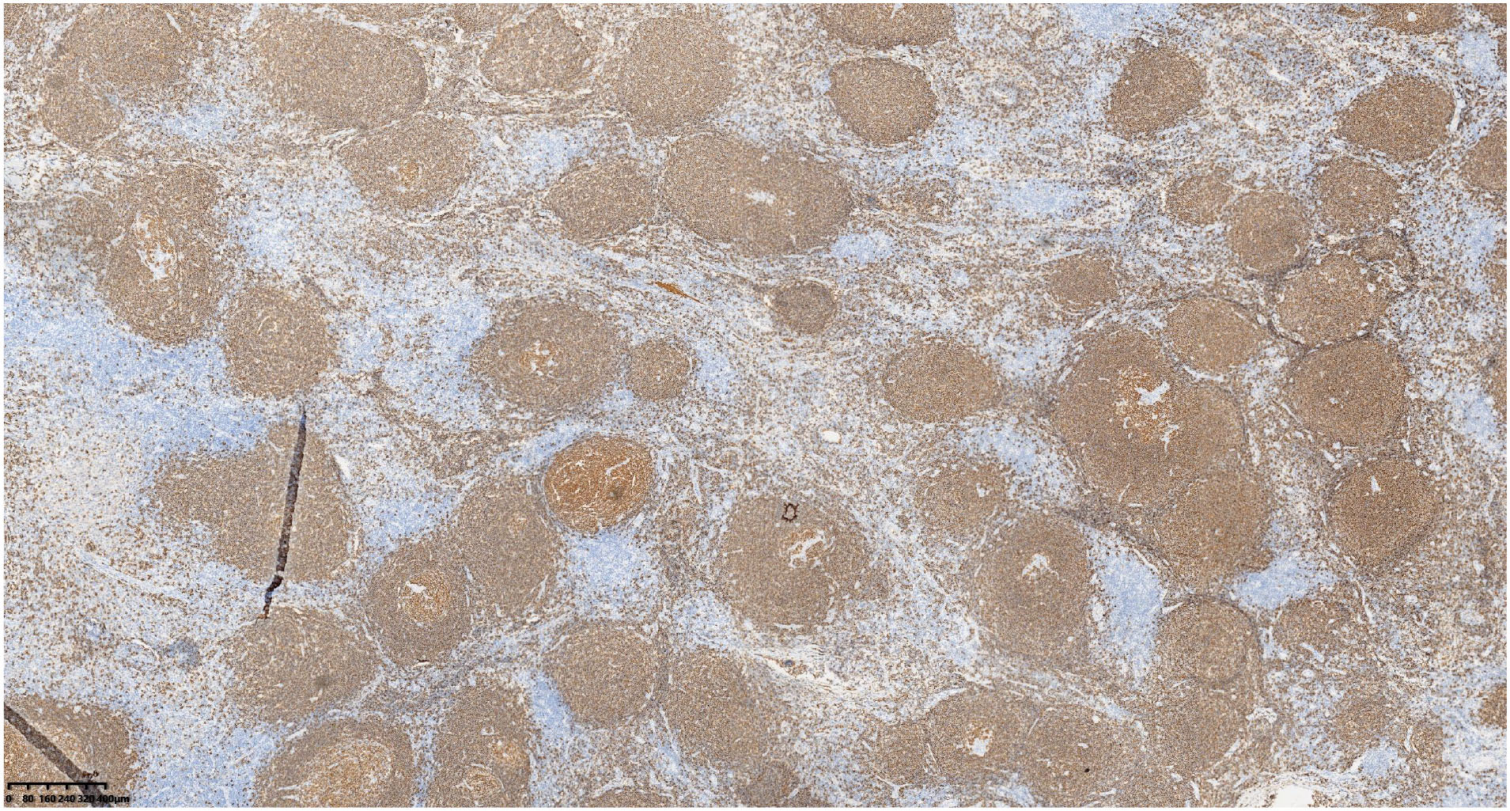
Lymphoid follicles are positive for CD20.

**Figure 6:**
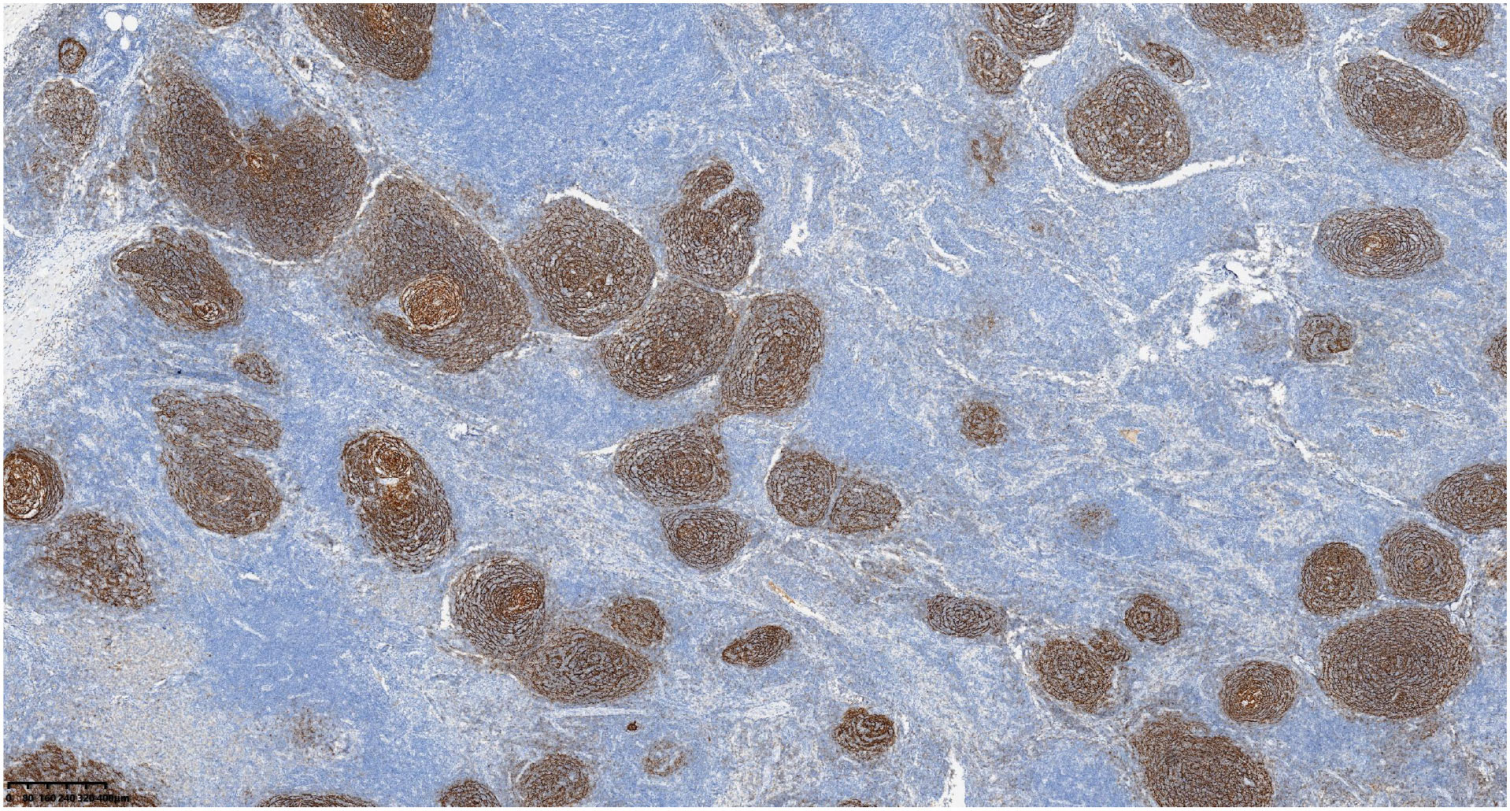
CD21 highlights preserved follicular dendritic cell (FDC) meshworks.

Postoperative pathological classification identified the hyaline vascular type in 9 patients, the plasma cell type in 1 patient, and the mixed type in 1 patient. Typical pathological images and immunohistochemical images are shown in **Figures 2**–**6**. The mean follow-up duration was 68.00 ± 30.41 months (range: 25–112 months). By the end of the follow-up period, no recurrence was detected in any patient, and all remained alive and healthy.Detailed information can be found in **Table 4**.

**Table 4:** Pathological results and prognosis of 11 patients with retroperitoneal UCD.

Case no.	Pathological type	Immunohistochemistry					Follow-up time (months)	Recurrence	Survival status
		CD3	CD20	CD10	Bcl-6	Ki-67			
1	Hyaline Vascular	+	+	–	–	10%	36	No	Alive
2	Hyaline Vascular	+	+	–	–	5%	55	No	Alive
3	Hyaline Vascular	+	+	+	+	10%	25	No	Alive
4	Hyaline Vascular	+	+	+	+	30%	42	No	Alive
5	Hyaline Vascular	+	+	–	–	10%	98	No	Alive
6	Hyaline Vascular	+	+	+	+	10%	86	No	Alive
7	Hyaline Vascular	+	+	+	+	30%	40	No	Alive
8	Plasma Cell	+	+	–	–	25%	112	No	Alive
9	Mixed	+	+	+	+	80%	107	No	Alive
10	Hyaline Vascular	+	+	+	+	20%	75	No	Alive
11	Hyaline Vascular	+	+	+	+	40%	72	No	Alive

## 4 Discussion

The pathogenesis of CD remains incompletely understood but may involve IL-6 overexpression or dysregulation of IL-6-related signaling pathways, potentially mediated by viruses such as HIV or HHV-8 (4–6). Previous reports indicate that CD occurring in the retroperitoneum is predominantly unicentric (7), which is consistent with our findings, as all 11 patients in this study had UCD.

Preoperative diagnosis of retroperitoneal UCD remains challenging. Firstly, regarding clinical symptoms, retroperitoneal UCD is often asymptomatic. In our study, 9 out of 11 cases were discovered incidentally during health check-ups. The remaining two patients presented due to mass effects (back/abdominal discomfort) caused by larger tumors. None of the patients exhibited systemic symptoms or elevated inflammatory markers beyond lymphadenopathy, and routine laboratory tests were unremarkable. Secondly, imaging findings on contrast-enhanced abdominal CT can vary, showing soft tissue masses with heterogeneous density and diverse enhancement patterns. The imaging characteristics often overlap with those of other retroperitoneal tumors such as paragangliomas, schwannomas, and leiomyosarcomas, leading to potential misdiagnosis (8). Furthermore, guidelines do not recommend fine-needle aspiration biopsy for retroperitoneal tumors (9). In summary, UCD lacks standard clinical symptoms, and its imaging findings are non-specific. Consequently, pathological examination following surgical resection remains the definitive diagnostic method. In 2010, Arlet et al. (10) established the gold standard for diagnosis, involving lymph node biopsy, pre- and postoperative pathological examination, and immunohistochemical studies. Histologically, characteristic features include lymphoid follicular hyperplasia, regressed germinal centers, prominent interfollicular vascular proliferation, and hyalinization penetrating the follicular centers, with concentric layering of lymphocytes in the mantle zone forming an “onion-skin” appearance. Thus, lymph node pathology is crucial for diagnosing CD and represents the gold standard. However, the typical pathological features can be limited, and the disease’s rarity often necessitates differentiation from other conditions, posing diagnostic challenges for pathologists and potentially requiring consultation with experienced specialists.

Pathologically, CD is classified into the hyaline vascular type (HV-CD), plasma cell type (PC-CD), and mixed type (11). Among patients with clinical UCD, the hyaline vascular type is predominant, accounting for 70%-90% of cases, and these patients rarely exhibit high inflammatory manifestations (1). In our study, 9 of 11 patients had the hyaline vascular type, and none showed high inflammatory signs, aligning with previous reports.

Complete surgical resection is the mainstay of treatment for UCD (3). Resectability should be assessed for all UCD patients. Whenever feasible, complete resection is the preferred approach, regardless of the presence of systemic symptoms. In our study, the mean tumor diameter was significantly larger than typical lymph node enlargement. This is likely attributable to the deep, spacious anatomical location of the retroperitoneum, allowing tumors to grow substantially before causing symptoms or being detected incidentally. Nevertheless, all patients in our series successfully underwent radical surgical resection.The operative times, blood loss, and hospital stays were manageable, with no severe postoperative complications, indicating that surgery for retroperitoneal UCD, despite often involving large tumors, can be performed safely with favorable outcomes.

Regarding follow-up, guidelines (3)recommend physical examination, biochemical tests, and CT imaging at 3 months postoperatively for asymptomatic UCD patients who have undergone complete resection, followed by annual assessments. All patients in our study were followed up. During follow-up, no patient required reoperation, rehospitalization, or died due to the tumor. There were no instances of severe long-term complications or radiological evidence of recurrence. Previous studies have reported that most UCD patients achieve long-term survival after complete resection, with a 5-year survival rate as high as 97.1% (12). Surgical resection for unicentric Castleman disease yields excellent results and long-term survival (13–15). Our prognostic data confirm that retroperitoneal UCD patients can be cured, with minimal recurrence and excellent prognosis, consistent with existing literature.

This study focused on UCD in the retroperitoneum, a distinctive location that prompts two noteworthy considerations. First, does retroperitoneal UCD possess unique characteristics compared to UCD at other sites? Based on our findings and literature review, several distinguishing features may exist: (1) Anatomy and Clinical Presentation: The spacious and deep-seated nature of the retroperitoneum often allows tumors to grow substantially before detection. In our series, the mean tumor diameter was considerable (5.23 cm), and most cases were incidental findings. This contrasts with UCD in more superficial or confined spaces like the neck or mediastinum, which tend to present earlier due to compression symptoms from even smaller masses. (2) Diagnostic Challenge: Its imaging features frequently overlap with other common retroperitoneal neoplasms (e.g., paraganglioma, schwannoma) (8). Furthermore, the deep location often precludes safe preoperative biopsy, rendering definitive diagnosis heavily reliant on postoperative pathology. (3) Surgical Considerations: The complex anatomy, with proximity to major vessels, urinary organs, and the gastrointestinal tract, may elevate the technical demand for complete resection, particularly for large or adherent tumors (e.g., the 10-cm lesion in our series). Crucially, however, once complete (R0) resection is achieved, the excellent long-term prognosis aligns with the established outcomes for UCD at all locations (3, 12).

Secondly, an intriguing observation from our series and much of the literature is that nearly all reported cases of isolated retroperitoneal CD are UCD, with MCD rarely reported to involve the retroperitoneum primarily. This phenomenon might be explained by the distinct pathogenesis of MCD, which is a systemic lymphoproliferative disorder often involving multiple superficial and deep nodal stations, driven by a pronounced systemic inflammatory response or cytokine storm (4). The retroperitoneal nodes, while part of the deep lymphatic system, may not be a predominant or characteristic “target site” for MCD. Alternatively, the diagnosis of MCD requires comprehensive systemic evaluation. An isolated retroperitoneal mass, without such an assessment, could be misclassified as UCD; however, the absence of disease development at other sites during long-term follow-up in all our patients supports their classification as true UCD. According to the systematic review by Talat et al., the UCD/MCD ratio was not clearly specified in surgical cases of retroperitoneal CD, warranting clarification by future large-scale studies.

It is important to acknowledge the limitations inherent in this study. First, its retrospective, single-center design introduces the potential for selection and information bias. Second, although our cohort size is substantial for such a rare entity, the small sample size limits the statistical power for more robust subgroup analyses (e.g., detailed comparison between surgical approaches or pathological subtypes) and precludes definitive generalizations. Third, due to the retrospective nature, comprehensive preoperative assessments such as PET-CT and systematic measurements of disease-related biomarkers (e.g., serum IL-6, IgG, HHV-8 status) were not uniformly available for all patients, which may affect the completeness of the clinical profile. Finally, as a study conducted at a tertiary referral center, the patient population may not be fully representative of all cases encountered in broader clinical practice. Despite these limitations, we believe that the detailed clinical, surgical, and long-term follow-up data presented provide valuable insights into the management of retroperitoneal UCD. Future prospective, multi-center studies with larger cohorts and standardized diagnostic protocols are warranted to validate our findings and further optimize the management strategy for this rare condition.

In conclusion, retroperitoneal unicentric Castleman disease is rare and lacks specific clinical manifestations. Complete surgical resection is the optimal treatment, associated with an excellent long-term prognosis. However, due to the rarity of the disease and the limited sample size in this study, future efforts should focus on systematic collection of cases and larger controlled studies to provide more robust scientific evidence for its diagnosis and treatment.

## Data Availability

The raw data supporting the conclusions of this article will be made available by the authors, without undue reservation.
